# Microwave dynamic therapy induces ferroptosis in colorectal cancer by targeting PTK2B to regulate STAT3-mediated GPX4 expression

**DOI:** 10.1186/s43556-025-00322-2

**Published:** 2025-10-30

**Authors:** Hui Zhou, Zijiang Zhang, Zhongtao Liu, Li Xiong, Xin Ran, Juan Liu, Yuping Ran, Yu Wen, Wei Chen, Jiachi Xu

**Affiliations:** 1 Department of General Surgery, The Second Xiangya Hospital of Central South University, Changsha, 410011 China; 2 School of Chips, XJTLU Entrepreneur College (Taicang), Xi’an Jiaotong-Liverpool University, Taicang, Taicang, Suzhou, Jiangsu 215400 China; 3 Department of Dermatovenereology, West China Hospital, Sichuan University, Chengdu, Sichuan Province China

**Keywords:** Colorectal cancer, MWDT, Ferroptosis, PTK2B, GPX4, STAT3

## Abstract

Colorectal cancer (CRC) is a common malignant tumor of the digestive tract, and chemotherapy resistance along with adverse reactions during treatment present significant challenges in clinical practice. Therefore, there is an urgent need to find effective and low-toxicity therapeutic agents. Ferroptosis, a recently discovered form of cell death, has attracted attention as a potential new cancer treatment strategy. Microwave dynamic therapy (MWDT), as a promising anticancer agent, has been shown to significantly inhibit various malignancies, including CRC. However, the role of MWDT in ferroptosis and its underlying mechanisms have not been fully explored. In this study, we demonstrated that MWDT treatment significantly increased levels of lipid peroxidation (LPO), reactive oxygen species (ROS), malondialdehyde (MDA), and Fe^2^⁺, while significantly decreasing glutathione (GSH) levels. Ferrostatin-1 partially inhibited these effects. Further research revealed that MWDT effectively inhibited tumor growth both in vitro and in vivo by inducing ferroptosis. At the molecular level, overexpression of PTK2B reversed the ferroptosis induced by MWDT. PTK2B interacted with STAT3 and enhanced its phosphorylation at Tyr705, promoting the nuclear translocation of p-STAT3 (Tyr705). In the nucleus, p-STAT3 bound to the GPX4 promoter region and promoted GPX4 transcription. MWDT inhibited PTK2B expression, suppressed STAT3 phosphorylation, and subsequently downregulated GPX4 transcription, thereby inducing ferroptosis. Our findings are the first to demonstrate that MWDT mediates ferroptosis in CRC via the PTK2B/STAT3/GPX4 axis, offering novel theoretical insights for its comprehensive treatment.

**Supplementary Information:**

The online version contains supplementary material available at 10.1186/s43556-025-00322-2.

## Introduction

Colorectal cancer (CRC) is a high-risk malignant tumor of the gastrointestinal tract [1]. Globally, CRC significantly impacts human health and quality of life, with elevated morbidity and mortality rates [1–3]. CRC cells have a propensity to spread to other parts of the body, including the Liver and lymph nodes. The risk of CRC increases with age, with the majority of patients typically diagnosed at 50 years or older [4]. However, in recent Years, CRC, especially rectal and distal colon cancer, has been on the rise in individuals under 50 years of age [5, 6]. Surgical resection remains the standard treatment for CRC, yet for some patients with advanced stages or metastases [7], surgery may not be optimal. Moreover, surgical resection may leave residual tumor cells, increasing the risk of metastasis [8]. Therefore, it is crucial to explore more effective treatments for advanced CRC to enhance patients' quality of life and survival rates.

Ferroptosis, a form of regulatory cell death characterized by iron-dependent lipid peroxidation, has emerged as a promising therapeutic target for cancer therapy [9]. It plays a significant role in eliminating various tumor cells, including colorectal cancer [10], pancreatic cancer [11], liver cancer [12], breast cancer [13] and ovarian cancer [14]. Dysregulation of ferroptosis impacts cell homeostasis and development, contributing to diseases like cancer [15]. Targeting ferroptosis offers a novel approach to cancer treatment as it promotes tumor inhibition. Molecules such as cystine/glutamate antiporter (System XC-), Glutathione peroxidase 4 (GPX4), Nuclear factor E2-related factor 2 (NRF2), acyl-CoA synthetase long-chain family member 4 (ACSL4), and solute carrier family 7a member 11 (SLC7A11) regulate ferroptosis by influencing iron metabolism and lipid peroxidation [16]. Recent studies have shown that induction of ferroptosis can effectively inhibit the proliferation and survival of cancer cells, providing a new way for the treatment of CRC [17].

Cysteamine copper (Cu-Cy), a novel photosensitizer, can be activated by light, X-rays, and microwaves to generate singlet oxygen for tumor treatment [18–20]. Cu-Cy offers several advantages, including a long half-life, low cytotoxicity, high singlet oxygen production, affordability, and ease of synthesis [21–24]. Chen et al. [25] demonstrated that X-PDT using X-ray-induced Cu-Cy effectively inhibits tumor cell proliferation and migration, confirming its efficacy in deep tumor models. However, X-ray irradiation for clinical cancer treatment poses significant risks to patients and healthy tissues, making it challenging to apply in practice. Microwave dynamic therapy (MWDT) mediated by microwave excitation of Cu-Cy emerges as a promising tumor treatment method [26]. Microwaves induce thermal effects during excitation, leading to blood vessel dilation, significantly increasing blood flow and oxygen perfusion [27, 28]. These factors can regulate hypoxia in the tumor microenvironment and promote the generation of reactive oxygen species (ROS), which has a therapeutic effect on tumors. Additionally, MW can overcome the depth penetration limitation of traditional photodynamic therapy, showing promise for deep tumor treatment. Our previous study demonstrated that Cu-Cy nanoparticles mediated MWDT effectively induced ferroptosis in cancer cells, offering a new clinical treatment option for cancer [26]. MWDT may serve as a potential treatment for drug-resistant and radioresistant cancers. However, the molecular mechanism of MWDT-induced ferroptosis in cancer cells remains unclear.

Protein tyrosine kinase 2 beta (PTK2B) is a non-receptor tyrosine kinase belonging to the focal adhesion kinase family [29]. Its activation is regulated by intracellular calcium, nicotinic acetylcholine receptor, membrane depolarization, or protein kinase C [30]. Notably, PTK2B can activate chloride channels in response to copper disulfiram stimulation, suggesting its role as a copper ion sensor [31]. Emerging evidence highlights PTK2B's significance in tumorigenesis and progression, acting as an oncogene in various cancers such as cervical cancer [32], and breast cancer [33], where its overexpression correlates with tumor progression and poor prognosis. However, PTK2B's role in CRC remains unclear.

In the present study, we investigated the molecular mechanism underlying MWDT-induced ferroptosis in CRC cells mediated by Cu-Cy. Our results showed that MWDT significantly reduced CRC cell viability and clone formation and promoted ferroptosis. In addition, we found that MWDT regulated the expression of GPX4, a key regulator of ferroptosis, through the PTK2B/STAT3 signaling axis. Specifically, we found that PTK2B regulates GPX4 transcription through activation of p-STAT3 (Tyr705). In addition, we provide evidence that PTK2B interacts with STAT3 and promotes its nuclear accumulation, further enhancing the induction of ferroptosis. Our findings suggest that MWDT not only reduces CRC cell proliferation but also triggers ferroptosis through the PTK2B/STAT3/GPX4 axis, providing a potential new therapeutic strategy for the treatment of CRC.

## Results

### MWDT reduces the viability and clonogenesis of CRC cells

**Fig. 1:**
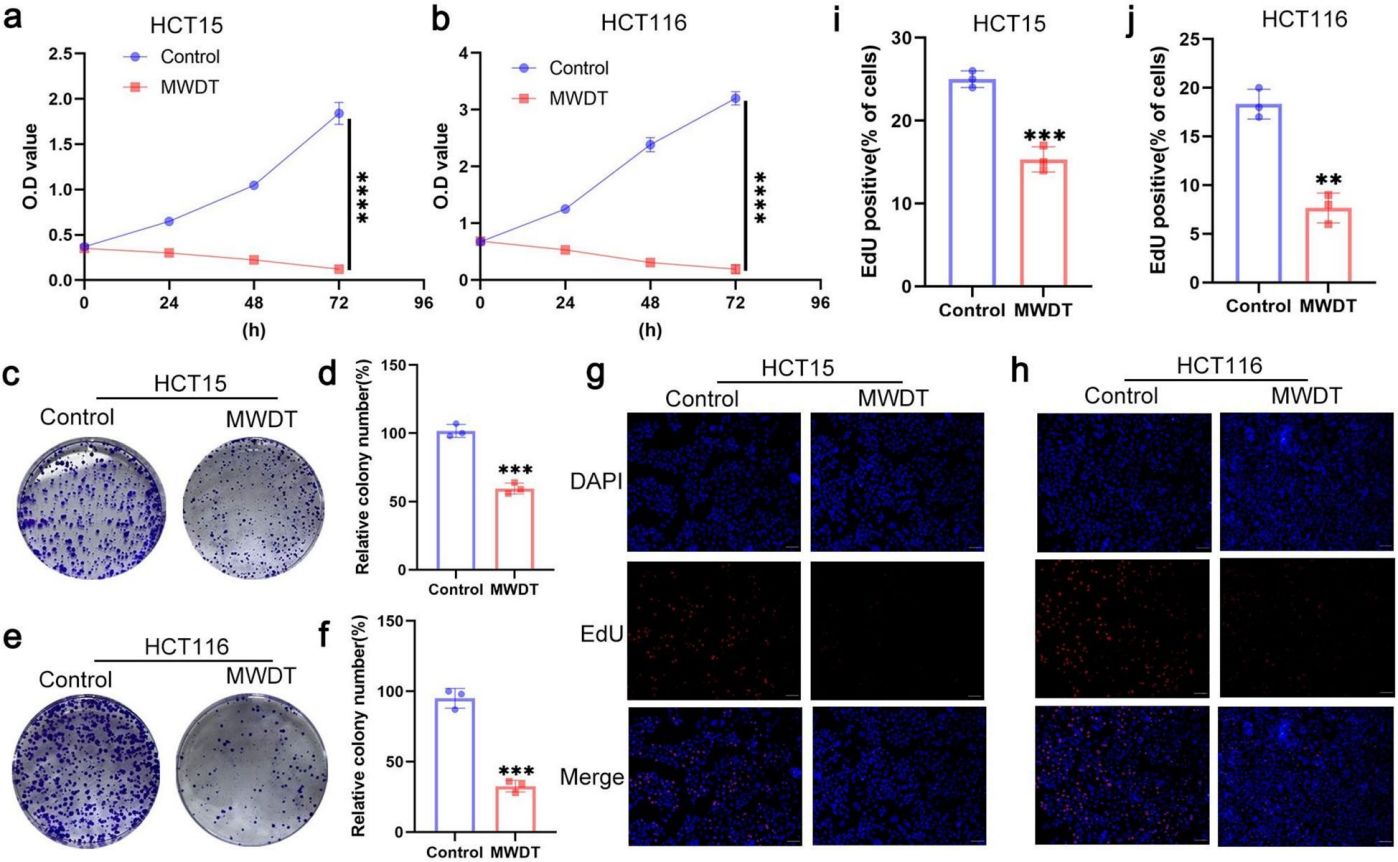
MWDT inhibits the proliferation of HCT15 and HCT116 cells. **a**, **b** The CCK-8 assay was used to measure the optical density (OD) values of CRC cells in different treatment groups at 0, 24, 48, and 72 h. **c**, **e** MWDT inhibited the growth of colonies in HCT15 and HCT116 cells. **d**, **f** Statistical analysis of colony-forming units. **g**, **h** The EdU proliferation assay was used to investigate the effect of MWDT on the proliferation of HCT15 and HCT116 cells. Scale bar, 100 μm. **i**, **j** Statistical analysis of the relative number of EdU-positive cells. ** *p* < 0.01 and ***p < 0.001

MWDT exhibits a strong anti-tumor effect, while CRC cells have a high proliferative capacity. Therefore, we first evaluated the inhibitory effect of MWDT on cell proliferation. The CCK-8 assay was used to assess the impact of MWDT on the growth of HCT15 and HCT116 cells. The results showed that MWDT significantly inhibited the proliferation of these cells (Fig. 1a, b). The colony formation assay further examined the long-term inhibitory effect of MWDT on HCT15 and HCT116 cell growth (Fig. 1c-f). The EdU proliferation assay was conducted to further evaluate the impact of MWDT on cell proliferation. The results demonstrated that MWDT significantly suppressed the proliferation of HCT15 and HCT116 cells (Fig. 1g-j). These findings collectively suggest that MWDT inhibits the proliferation of CRC cells.

### MWDT induces ferroptosis in CRC cells

**Fig. 2:**
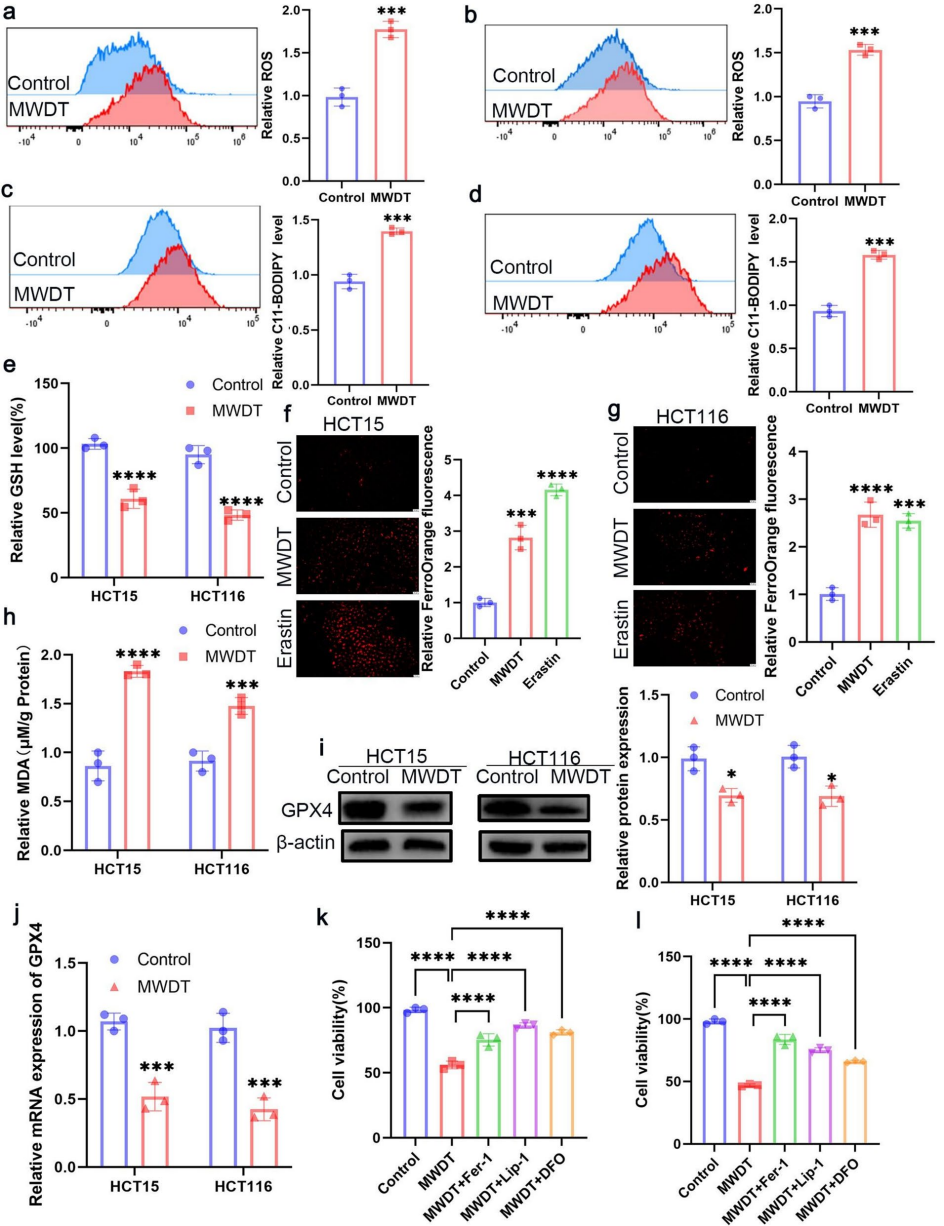
MWDT induces ferroptosis of HCT15 and HCT116 cells. **a**, **b** Flow cytometry analysis of ROS levels in HCT15 and HCT116 cells treated with MWDT. Significant increases in ROS were observed in both cell lines upon MWDT treatment compared to the control group. **c**, **d** Flow cytometry analysis of C11-BODIPY levels, indicating LPO in HCT15 and HCT116 cells. MWDT treatment significantly increased lipid peroxidation. **e** Relative levels of GSH in HCT15 and HCT116 cells treated with MWDT, showing a significant decrease in GSH levels upon treatment. **f**, **g** FerroOrange fluorescence imaging in HCT15 (**f**) and HCT116 (**g**) cells treated with MWDT. The statistical results of the relative FerroOrange fluorescence of CRC cells. **h** Relative MDA levels in HCT15 and HCT116 cells. MWDT treatment resulted in a significant increase in MDA levels. **i** Western blot analysis of GPX4 protein expression in HCT15 and HCT116 cells. **j** Relative mRNA expression of GPX4 in HCT15 and HCT116 cells. **k**, **l** Cell viability assays showing the effects of MWDT on cell viability in HCT15 (**k**) and HCT116 (**l**) cells in the presence of various conditions: MWDT, MWDT + Fer-1(1 μM), MWDT + Lip-1(1 μM), and MWDT + DFO (5 μM). *P < 0.05, ***P < 0.001, ****P < 0.0001

CRC is relatively insensitive to erastin, a classic ferroptosis inducer, suggesting that CRC exhibits inherent resistance to ferroptosis induction [34]. To evaluate whether MWDT can induce ferroptosis in CRC cells, we systematically investigated key features of ferroptosis, including intracellular ROS accumulation, lipid peroxidation levels, Fe^2+^concentrations, glutathione (GSH) levels, and expression changes in ferroptosis-related proteins. Initially, we assessed ROS levels using the DCFH-DA fluorescence probe combined with flow cytometry. The results demonstrated that MWDT treatment significantly promoted ROS accumulation in CRC cells (Fig. 2a, b). To further validate lipid peroxidation, we employed the C11-BODIPY probe to measure lipid ROS levels. Our findings revealed that MWDT treatment significantly increased lipid peroxidation (Fig. 2c, d). Additionally, MWDT treatment led to a substantial decrease in intracellular GSH levels (Fig. 2e), a crucial antioxidant molecule in cells, the depletion of which is a hallmark of ferroptosis. We also assessed malondialdehyde (MDA) levels, a final product of lipid peroxidation. The results indicated that MWDT significantly upregulated MDA levels in CRC cells (Fig. 2h). To investigate the effect of MWDT on iron metabolism, we used the FerroOrange probe to detect intracellular Fe^2+^ levels. Confocal fluorescence imaging revealed that MWDT treatment significantly enhanced Fe^2+^ accumulation in CRC cells (Fig. 2f, g). Furthermore, we analyzed the expression levels of GPX4 (glutathione peroxidase 4) through qPCR and Western blot analysis. GPX4 is a key inhibitor of ferroptosis, primarily responsible for eliminating lipid peroxides on cell membranes. Our results demonstrated that MWDT treatment significantly reduced both the mRNA and protein levels of GPX4 (Fig. 2i, j). Lastly, to further confirm that the cell death induced by MWDT was ferroptosis-dependent, we performed intervention experiments using three ferroptosis-specific inhibitors: ferrostatin-1(Fer-1), deferoxamine (DFO), and liproxstatin-1(Lip-1). These inhibitors effectively blocked MWDT-induced cell death in CRC cells (Fig. 2k, l). In summary, our findings indicate that MWDT triggers ferroptosis in CRC cells by inducing ROS accumulation, LPO, Fe^2+^ accumulation, and downregulation of GPX4 expression. These results provide significant experimental evidence for MWDT as a potential therapeutic strategy.

### MWDT inhibits cancer cell proliferation by regulating ferroptosis

**Fig. 3:**
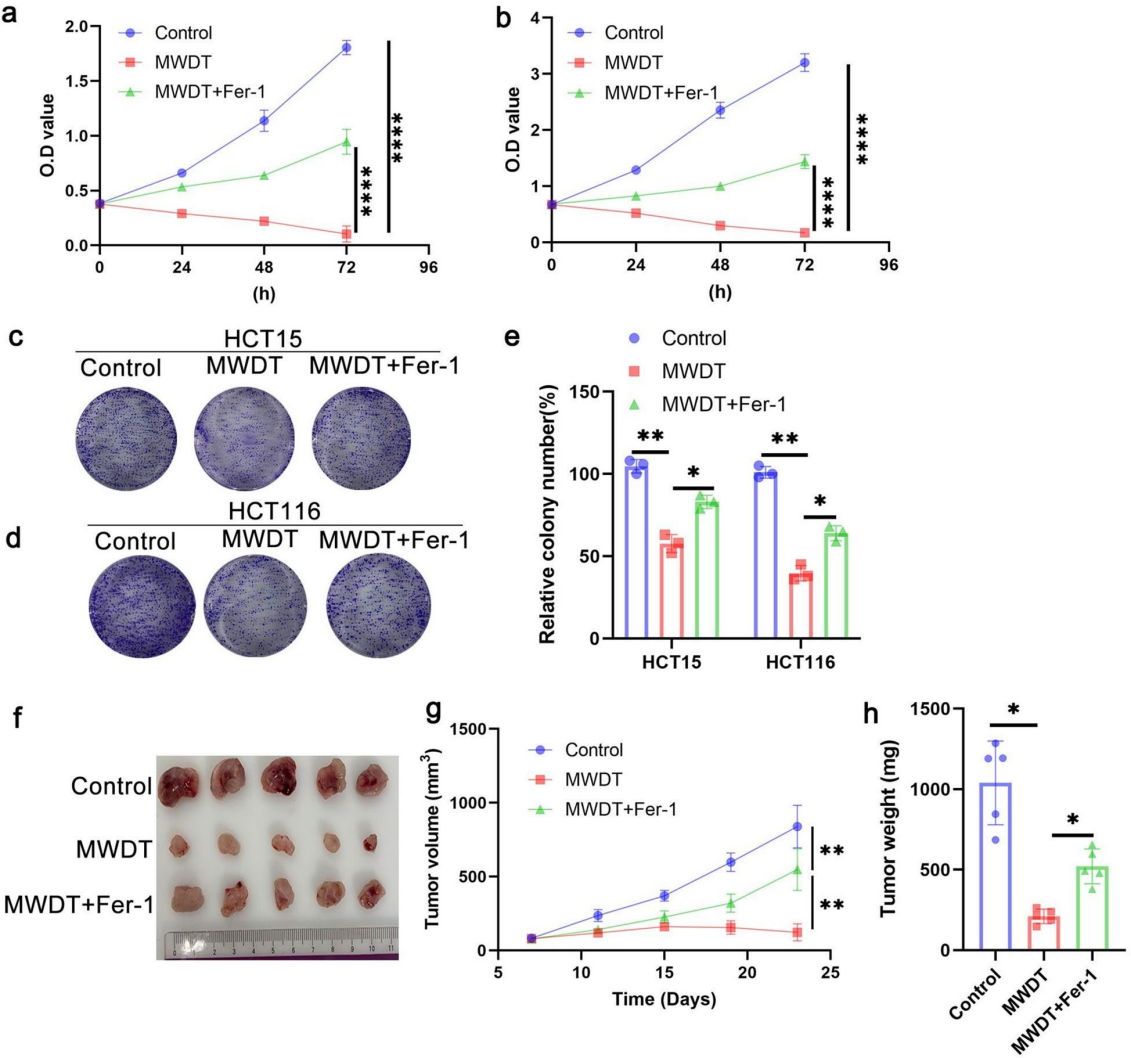
MWDT inhibits cancer cell proliferation by regulating ferroptosis. **a**, **b** O.D. values of HCT15 and HCT116 cells after treatment with Control, MWDT, or MWDT + Fer-1 at 0, 24, 48, and 72 h. **c**, **d** Representative colony formation assays in HCT15 and HCT116 cells after 14 days. **e** Quantification of colony numbers in HCT15 and HCT116 cells. **f** Tumor images from HCT116 xenografts. **g** The tumor volume changes were monitored and presented for animals undergoing different treatments. **h** Tumor weight measurement following different treatments. *p < 0.05, **p < 0.01 and ****P < 0.0001

To verify whether MWDT inhibits cell proliferation through inducing ferroptosis, we used the ferroptosis inhibitor Fer-1 as an experimental intervention. The cells were divided into three groups (Control, MWDT, and MWDT + Fer-1), and cell proliferation was observed for each group. MWDT significantly reduced the proliferation of HCT15 and HCT116 cells, while Fer-1 significantly rescued this inhibition (Fig. 3a, b). We then performed colony formation assays on the three groups of cells. The results confirmed that MWDT significantly inhibited the clonogenicity of pancreatic cancer cells, while Fer-1 rescued this effect (Fig. 3c-e). We next evaluated the in vivo relevance of this mechanism using a xenograft model. HCT116 cells were injected into nude mice to form subcutaneous tumors. Once the tumors became palpable, the mice were treated with MWDT, and Fer-1 (5 mg/kg) was administered intraperitoneally every four days. Throughout the period, tumor growth was regularly observed, and tumor size was measured and recorded weekly. After 23 days, the mice were euthanized, and tumors were excised and weighed. The results showed that MWDT inhibited tumor growth, while the ferroptosis inhibitor Fer-1 reversed this effect (Fig. 3f-h). These data thus confirm the in vivo efficacy of MWDT for CRC treatment.

### MWDT modulates GPX4 expression via PTK2B to regulate ferroptosis

**Fig. 4:**
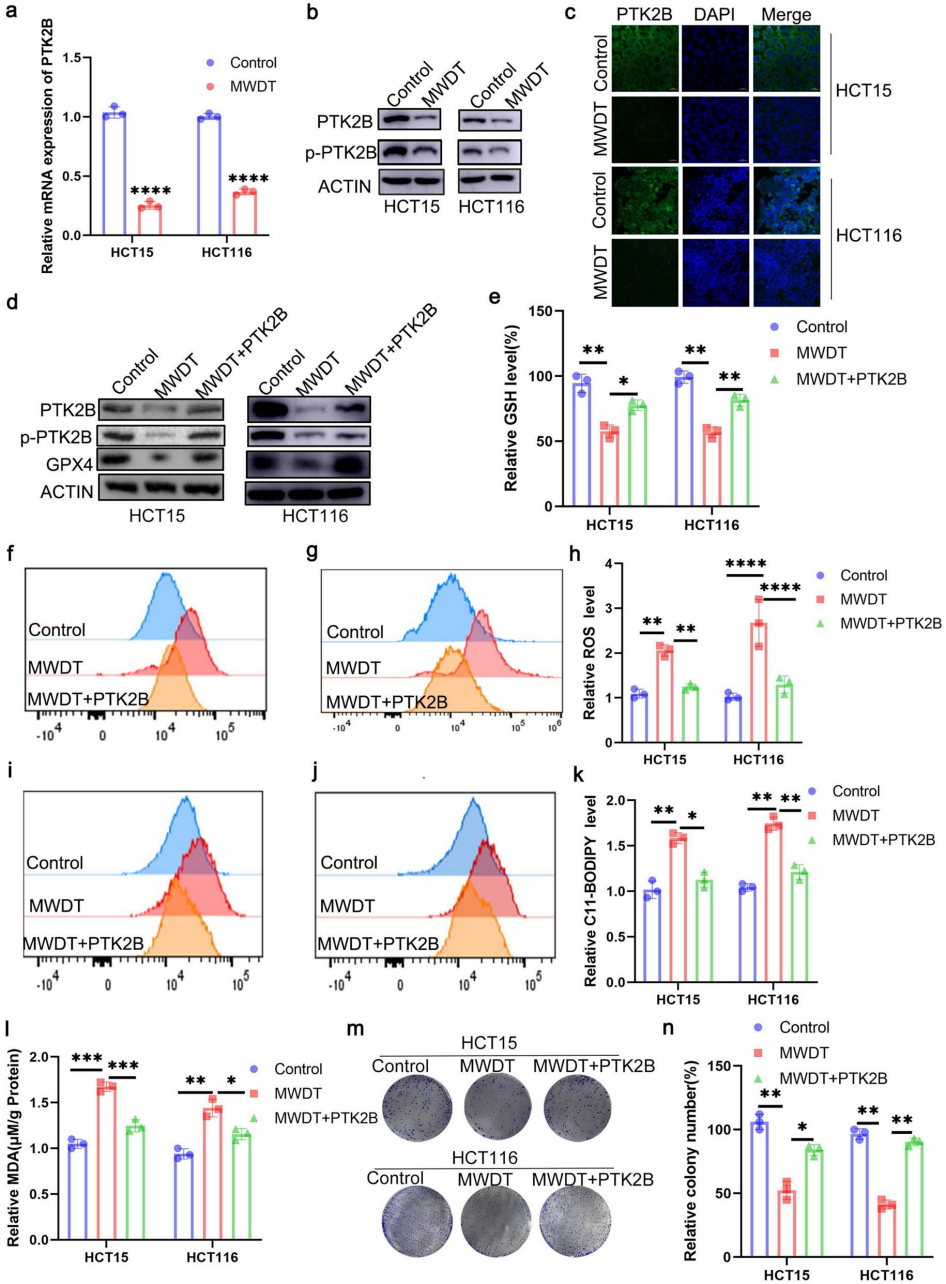
MWDT modulates GPX4 expression via PTK2B to regulate ferroptosis. **a** Relative mRNA expression levels of PTK2B in HCT15 and HCT116 cells treated with MWDT, with and without PTK2B inhibition. **b** Western blotting analysis of PTK2B levels in HCT15, and HCT116 cells receiving different treatments. **c** Immunofluorescence assay showed that the expression of PTK2B was decreased in the MWDT group compared with the empty vector group. **d** Western blotting analysis of PTK2B, P-PTK2B, and GPX4 levels in HCT15, and HCT116 cells receiving different treatments. **e** The relative levels of GSH were measured in HCT15 and HCT116 cells treated with MWDT and PTK2B inhibition. **f**–**h** Flow cytometry analysis of ROS levels indicated a marked increase in ROS in both HCT15 and HCT116 cells treated with MWDT, which was reversed by PTK2B inhibition. **i**-**k** Flow cytometry analysis of C11-BODIPY levels indicated a marked increase in LPO in both HCT15 and HCT116 cells treated with MWDT, which was reversed by PTK2B inhibition. **l** MDA levels were significantly elevated in MWDT-treated cells, with a marked decrease observed in the PTK2B inhibition group. **m**, **n** Representative colony formation images (**m**) and quantification (**n**) of HCT15 and HCT116 cells show that PTK2B inhibition mitigates the colony formation ability of CRC cells treated with MWDT. *P < 0.05, **P < 0.01, ***P < 0.001, and ****P < 0.0001

Recent studies have found that DSF/Cu complex induces the death of prostate cancer cells through the activation of CLC3 chloride channel with the participation of PTK2B [31]. This finding prompted us to further consider the potential role of PTK2B in MWDT. Therefore, we hypothesize that MWDT may promote ferroptosis through PTK2B, which may provide a new molecular mechanism for cancer treatment. To test this hypothesis, the expression levels of PTK2B mRNA in HCT15 and HCT116 cells treated with MWDT were measured by real-time quantitative PCR. The results demonstrated a notable decrease in PTK2B mRNA levels 24 h after MWDT treatment (Fig. 4a). Western blot analysis further confirmed that MWDT significantly suppressed PTK2B protein expression, accompanied by a corresponding decrease in its phosphorylation (Fig. 4b). Additionally, immunofluorescence staining showed a reduction in PTK2B expression in MWDT-treated cells (Fig. 4c). To further explore whether the induction of ferroptosis by MWDT is linked to PTK2B downregulation, PTK2B was overexpressed in HCT15 and HCT116 cells, which were then treated with MWDT. Western blot and qPCR results showed that MWDT treatment led to the downregulation of GPX4, and overexpression of PTK2B partially reversed the GPX4 downregulation (Fig. 4d). Furthermore, MWDT-induced decreases in GSH levels and increases in MDA, ROS, and LPO levels were partially reversed following PTK2B overexpression (Fig. 4e-l). In colony formation assays, overexpression of PTK2B partially counteracted the inhibitory effect of MWDT on tumor cell proliferation (Fig. 4m, n). In conclusion, these findings highlight that MWDT exerts its anti-cancer effects by modulating the PTK2B/GPX4 signaling pathway.

### PTK2B regulates GPX4 transcription through p**‑**STAT3 (Tyr705)

**Fig. 5:**
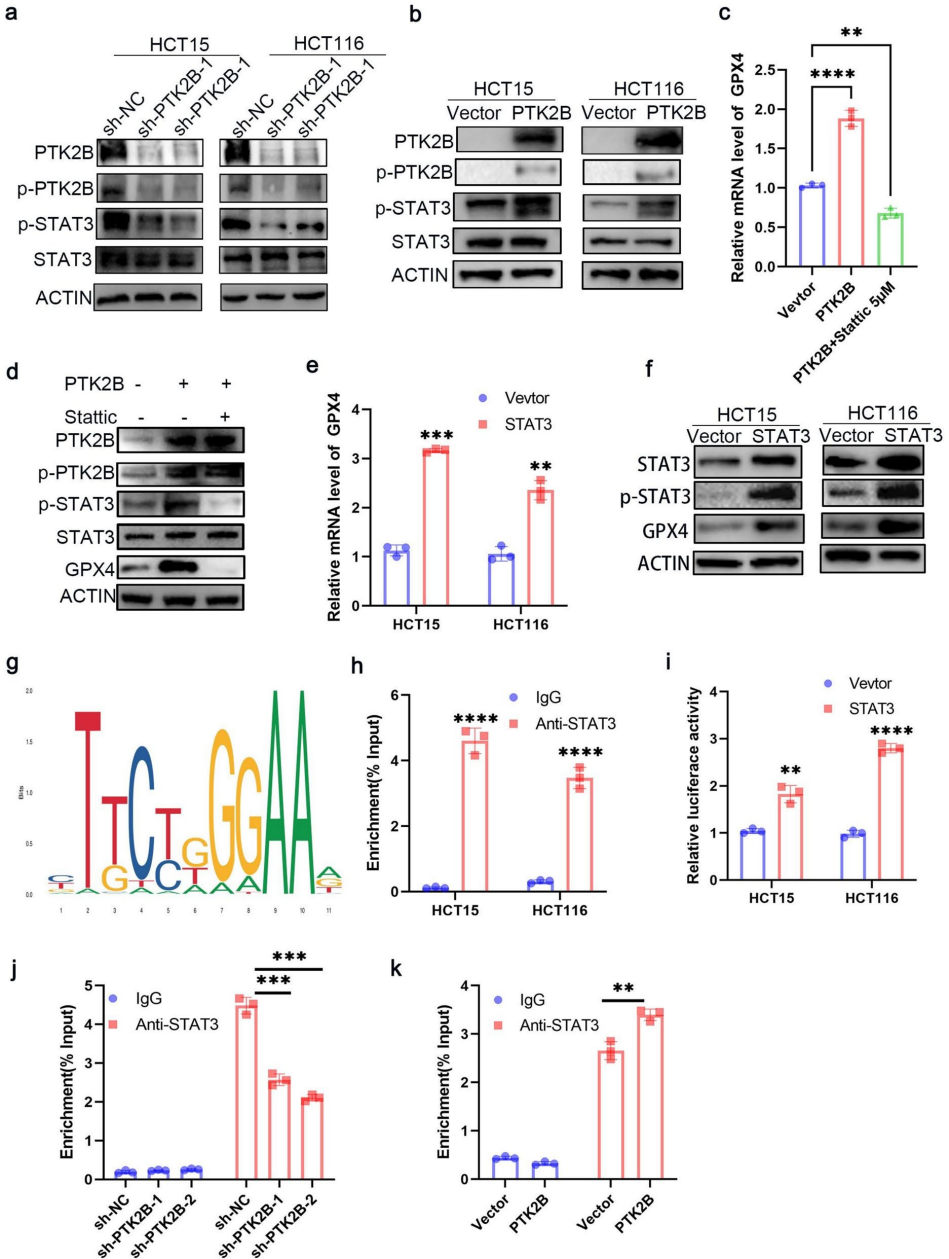
PTK2B regulates GPX4 transcription through p-STAT3 (Tyr705). **a**, **b** PTK2B positively regulated the phosphorylation of STAT3 at Tyr705 in HCT15, and HCT116 cells. **c** The mRNA expression of GPX4 in HCT15 PTK2B overexpression cell lines, with or without treatment with Stattic (5 µM). **d** Protein levels of PTK2B, p-PTK2B, STAT3, p-STAT3, and GPX4 in HCT15 PTK2B overexpression cell lines, with or without treatment with Stattic (5 µM). **e**,**f** Western blotting and qRT-PCR analyses of the change in GPX4 expression caused by STAT3 overexpression in CRC cells. **g** JASPAR predicted a conserved STAT3 binding motif, and a schematic diagram of the potential STAT3 binding site in the GPX4 promoter is shown in the figure. **h** ChIP analysis of STAT3 occupancy at the GPX4 promoter in HCT15 cells and HCT116 cells. **i** Luciferase reporter assays were conducted in HCT15 and HCT116 cells overexpressing STAT3 and GPX4 reporter plasmids. **j**-**k** ChIP-qPCR analysis was performed using anti-STAT3 antibody or control IgG in HCT15 cells with stable PTK2B knockdown or PTK2B overexpression.**p < 0.01, ***P < 0.001, and ****P < 0.0001

The above results indicate that PTK2B promotes GPX4 mRNA expression; however, given its nature as a cytoplasmic tyrosine kinase, it is likely to regulate GPX4 expression indirectly via other transcriptional regulators. Among the potential downstream effectors, STAT3 has been reported to modulate GPX4 transcription in pancreatic cancer cells [35]. We therefore hypothesized that PTK2B might regulate GPX4 expression via a STAT3-dependent transcriptional mechanism. We examined total STAT3 and p-STAT3 (Tyr705) levels in HCT15 and HCT116 cells with stable PTK2B knockdown, as well as in HCT15 cells with stable PTK2B overexpression. PTK2B silencing decreased the phosphorylation of STAT3 at Tyr705, whereas PTK2B overexpression increased it; however, total STAT3 levels remained unchanged (Fig. 5a,b). In the HCT15 PTK2B-overexpressing cell line, treatment with the p-STAT3 (Tyr705) inhibitor Stattic (5 µM) reversed the PTK2B-induced increases in GPX4 mRNA and protein levels (Fig. 5c,d).

We next investigated how STAT3 regulates GPX4 expression. Overexpression of STAT3 in HCT15 cells upregulated GPX4 mRNA and protein levels (Fig. 5e,f). To explore whether STAT3 directly regulates GPX4 transcription, we first analyzed published STAT3 chromatin immunoprecipitation sequencing (ChIP-seq) datasets using the Cistrome Data Browser (http://db3.cistrome.org/browser), which revealed STAT3-binding peaks within the GPX4 promoter region in SU-DHL2 and OCI-Ly10 cells (Fig. S1). Using the JASPAR database (http://jaspar.genereg.net/), we predicted STAT3-binding sites in the GPX4 promoter (Fig. 5g). Chromatin immunoprecipitation followed by qPCR (ChIP-qPCR) demonstrated significant enrichment of primers targeting the GPX4 promoter region (Fig. 5h).

To further confirm the transcriptional regulation of GPX4 by STAT3, we cloned the GPX4 promoter into a dual-luciferase reporter construct and transfected it into HCT15 and HCT116 cells. Luciferase reporter assays showed that STAT3 overexpression significantly enhanced GPX4 promoter activity in both cell lines (Fig. 5i). To determine whether PTK2B modulates STAT3 occupancy at the GPX4 promoter, ChIP-qPCR assays revealed that PTK2B silencing reduced, whereas PTK2B overexpression increased, STAT3 binding affinity to the GPX4 promoter (Fig. 5j, k).Collectively, these findings demonstrate that PTK2B regulates GPX4 transcription by modulating p-STAT3 (Tyr705) levels, thereby influencing GPX4 expression.

### PTK2B regulates STAT3 phosphorylation and directly interacts with STAT3

**Fig. 6:**
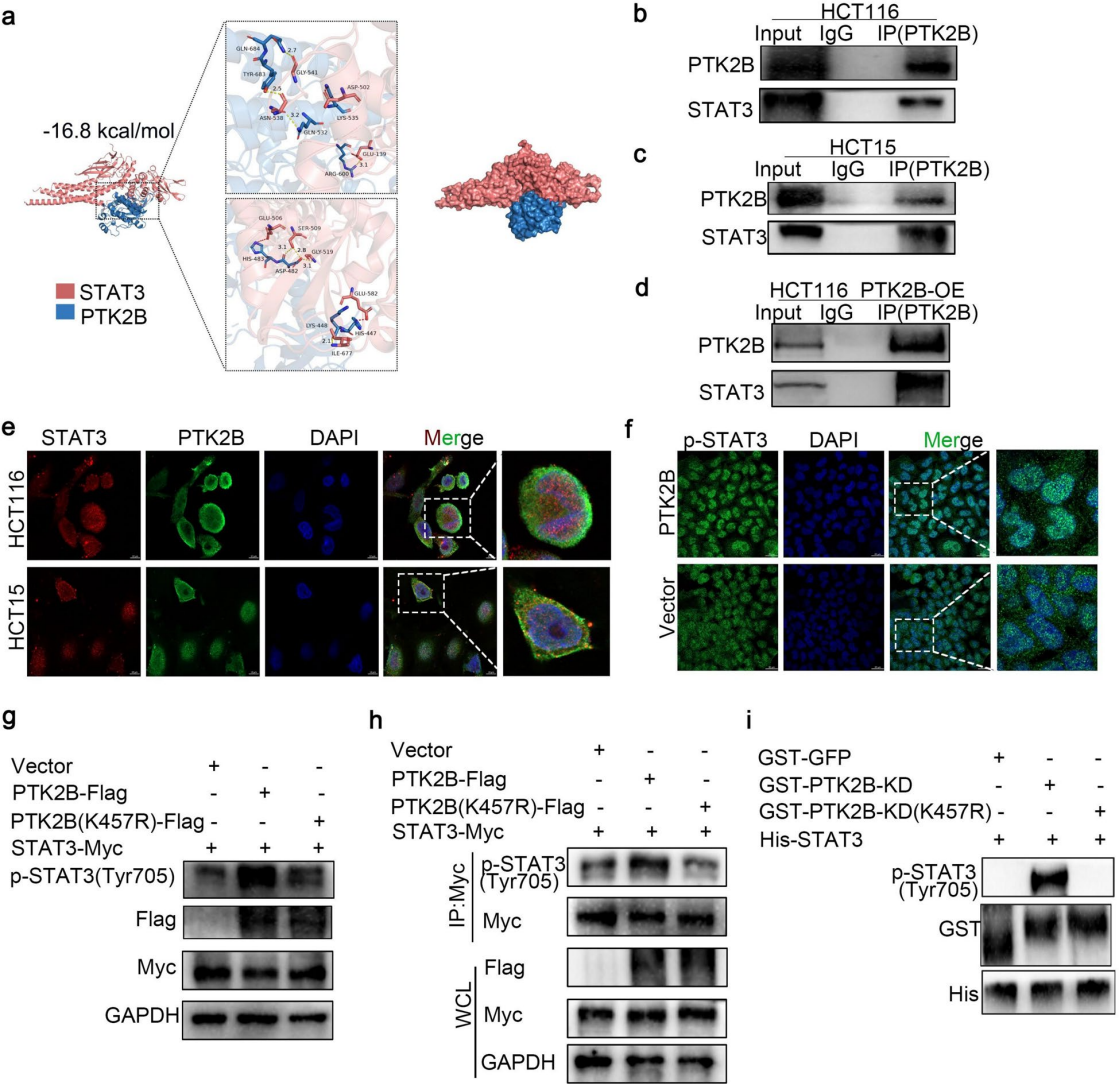
p-PTK2B(Tyr402) interacts with STAT3 and promotes nuclear accumulation of p-STAT3 (Tyr705). **a** Molecular docking analysis showing a strong binding effect between PTK2B and STAT3. **b**-**d** Co-IP assays showing the interaction between PTK2B and STAT3 in HCT116, HCT15, and HCT116 PTK2B overexpression cells. Blots were probed for PTK2Band STAT3. **e** IF staining showing co-localization of STAT3 (red) and PTK2B (green) in HCT116 and HCT15 cells. Nuclei are stained with DAPI (blue). **f** IF analysis showing nuclear accumulation of p-STAT3 (Tyr705) in HCT116 cells overexpressing PTK2B or vector. p-STAT3 (Tyr705) is shown in green and DAPI in blue. **g** Western blot analysis showing that overexpression of wild-type PTK2B significantly enhances phosphorylation of STAT3 at tyrosine 705, whereas kinase-inactive PTK2B (K457R) does not. **h** Co-IP of STAT3-Myc, followed by phosphorylation detection of STAT3 at Tyr705, showing that wild-type PTK2B promotes STAT3 phosphorylation. **i** In vitro kinase assay showing that GST-PTK2B kinase domain (GST-PTK2B-KD) efficiently phosphorylates STAT3 at Tyr705, while the K457R mutant does not

The results showed that PTK2B positively regulated the expression of p-STAT3 (at tyrosine 705) but had no significant effect on the total STAT3 level (Fig. 5b). To further explore how PTK2B regulates STAT3 function, we performed a more in-depth analysis. We performed molecular docking by using PyMOL software, and the results showed that the binding energy of PTK2B to STAT3 was −16.8 kcal/mol, which showed a strong binding effect (Fig. 6a). We further investigated the interaction between PTK2B and STAT3 by co-immunoprecipitation. The results showed that PTK2B interacted with STAT3 in HCT15 and HCT116 cells (Fig. 6b, c). Next, we transiently transfected HCT116 cells with a FLAG-tagged PTK2B expression plasmid and performed coimmunoprecipitation using anti-FLAG antibody. The results showed that the interaction between PTK2B and STAT3 was enhanced in these cells (Fig. 6d). To further verify this interaction, immunofluorescence experiments were performed to analyze the colocalization of PTK2B and STAT3. Immunofluorescence results showed that PTK2B and STAT3 were co-localized in HCT15 and HCT116 cells (Fig. 6e). In addition, immunofluorescence experiments also confirmed that overexpression of PTK2B promoted the intranuclear aggregation of p-STAT3 (Tyr705) (Fig. 6f). To explore whether PTK2B is able to directly phosphorylate STAT3, we transfected STAT3-Myc into HEK293T cells together with wild-type PTK2B or a kinase inactivating mutant, PTK2B-K457R. As shown in Fig. 6g, overexpression of wild-type PTK2B significantly enhanced STAT3 phosphorylation at tyrosine 705, whereas kinase-inactivated PTK2B mutants failed to produce this effect. To confirm that STAT3 is a substrate of PTK2B, we performed a coimmunoprecipitation assay. STAT3-Myc was immunoprecipitated using an anti-Myc antibody, and the phosphorylation of tyrosine 705 was subsequently measured. Consistent with the above results, STAT3 phosphorylation at tyrosine 705 was significantly increased in the presence of wild-type PTK2B but not the K457R mutant (Fig. 6h), providing further evidence that PTK2B promotes STAT3 phosphorylation. To determine whether PTK2B directly phosphorylated STAT3, we performed in vitro kinase activity assay experiments using purified GST-PTK2B kinase domain (GST-PTk2B-KD) and STAT3 with His tag (His-STAT3). As shown in Fig. 6i, GST-PTK2B-KD (but not its kinase-inactive variant K457R) was able to efficiently phosphorylate STAT3 at Tyr705 in vitro, a result confirmed by an antibody that specifically detects phosphorylated STAT3(Tyr705). These results suggest that PTK2B directly phosphorylates STAT3 at Tyr705 in a manner dependent on its kinase activity.

### MWDT regulates the transcription of GPX4 by modulating the PTK2B-STAT3 axis

**Fig. 7:**
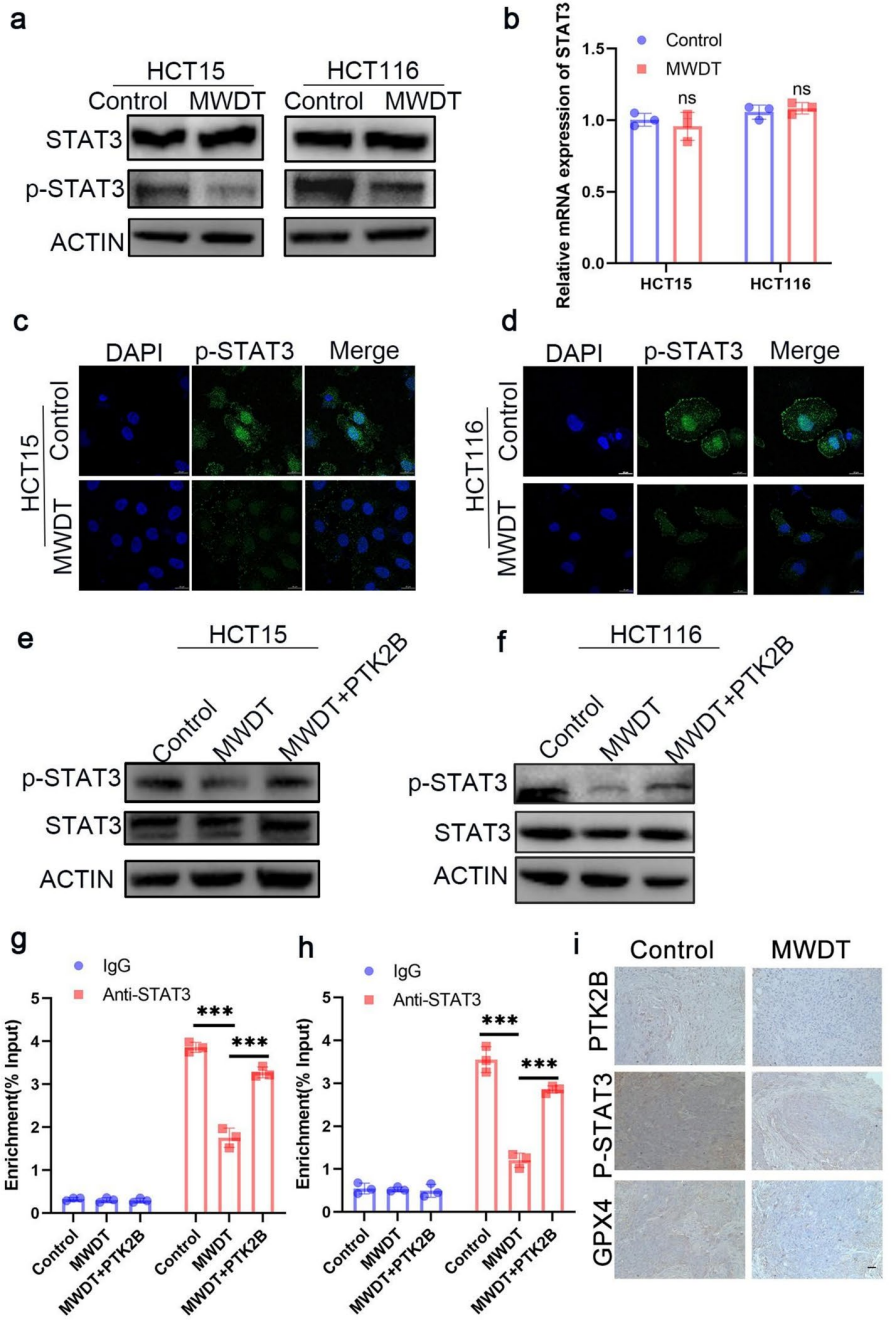
MWDT regulates the transcription of GPX4 by modulating the PTK2B-STAT3 axis. **a** MWDT treatment significantly decreased the phosphorylation of STAT3 at Tyr705 in HCT15 and HCT116 cells, without altering total STAT3 protein levels. **b** Relative mRNA expression levels of STAT3 in HCT15 and HCT116 cells treated with MWDT. **c**,**d** Immunofluorescence staining reveals a significant reduction in nuclear p-STAT3 accumulation following MWDT treatment in HCT15 cells and HCT116 cells. **e**,**f** Western blot analysis of p-STAT3 and total STAT3 protein levels in HCT15 cells and HCT116 cells treated with MWDT, with or without PTK2B overexpression. **g**,**h** ChIP-qPCR results showing reduced binding of STAT3 to the GPX4 promoter in MWDT-treated cells, an effect partially alleviated by PTK2B overexpression. **i** Immunohistochemical staining for PTK2B, P-STAT3, and GPX4 in tumors. Scale bar: 50 μm

To determine whether MWDT affects the activation of STAT3, we examined both the total protein level of STAT3 and its phosphorylated form at tyrosine 705. Western blot analysis revealed that MWDT significantly reduced the levels of p-STAT3 (tyrosine 705) in HCT15 and HCT116 cells, but did not alter the total STAT3 protein expression (Fig. 7a). Similarly, qRT-PCR analysis showed that MWDT had no effect on the mRNA expression of STAT3 (Fig. 7b), suggesting that MWDT may inhibit the activation of STAT3 at the post-translational level. Immunofluorescence staining further confirmed that MWDT significantly decreased the accumulation of p-STAT3 (tyrosine 705) in the nucleus (Fig. 7c, d), indicating that its nuclear translocation was inhibited.

To investigate whether this inhibitory effect was mediated by PTK2B, we overexpressed PTK2B in cells treated with MWDT. Notably, the overexpression of PTK2B partially restored the level of p-STAT3 under MWDT treatment (Fig. 7e, f), indicating that PTK2B acts upstream of STAT3 activation. ChIP-qPCR analysis showed that MWDT significantly reduced the binding of STAT3 to the GPX4 promoter, while PTK2B overexpression partially alleviated this effect (Fig. 7g, h), further supporting the idea that MWDT inhibits STAT3 transcriptional activity by targeting PTK2B. Finally, immunohistochemical analysis of xenograft tumor tissues showed that MWDT treatment in vivo reduced the expression of PTK2B, p-STAT3, and GPX4 (Fig. 7i), confirming the inhibition of this signaling pathway within the tumor microenvironment.

Taken together, these findings suggest that MWDT inhibits the expression of PTK2B, thereby reducing the phosphorylation and nuclear localization of STAT3, which in turn decreases its binding to the GPX4 promoter and suppresses the transcription of GPX4, ultimately promoting ferroptosis induction.

## Discussion

Despite the continuous emergence of treatment strategies such as chemotherapy and immunotherapy, the prognosis of CRC has significantly improved; however, CRC remains a major cause of cancer-related deaths globally [7]. A substantial proportion of patients fail to benefit from conventional treatments or experience relapse, underscoring the urgent need to explore alternative therapeutic strategies and their underlying mechanisms. Recent studies suggest that inducing ferroptosis could serve as an effective strategy to eliminate drug-resistant cancers [36, 37], while CRC exhibits inherent resistance to ferroptosis induction [34]. Therefore, exploring treatment strategies that induce ferroptosis in CRC holds significant long-term potential.

In this study, we investigated the potential of MWDT in inducing ferroptosis in CRC cells and explored its cancer therapeutic potential and molecular mechanisms. Our results show that MWDT significantly reduced CRC cell viability and clonogenicity. Specifically, MWDT treatment led to ferroptosis in CRC cells. The ferroptosis inhibitor Ferrostatin-1 partially reversed this effect, further supporting the idea that MWDT induces ferroptosis through an iron-dependent mechanism. Mechanistically, we innovatively discovered that MWDT regulates the expression of GPX4 via influencing PTK2B, thereby impacting ferroptosis. We demonstrated that activated PTK2B interacts with STAT3 and promotes its phosphorylation at the Tyr705 site, facilitating the nuclear accumulation of p-STAT3. In the nucleus, p-STAT3 binds to the GPX4 promoter and enhances its transcription. GPX4 is a critical regulator of ferroptosis, and its expression is vital for protecting cells from lipid peroxidation. Therefore, by modulating PTK2B and STAT3, MWDT indirectly downregulated GPX4 expression and induced ferroptosis in CRC cells.

Recent advancements in novel nanomaterials for cancer therapy have shown remarkable progress. Notably, Cu(I)-based nanoparticles can generate more hydroxyl radicals (·OH) in the weakly acidic tumor microenvironment [37]. Additionally, excess GSH in the tumor microenvironment can reduce Cu(II) to Cu(I), further accelerating the reaction rate [38]. Our previous studies have confirmed that Cu-Cy, a novel Cu(I)-ketone photosensitizer, can generate ROS under conventional light (e.g., ultraviolet), as well as under ultrasound, X-ray, or microwave exposure [22, 26]. It is effective not only for treating superficial and deep cancers but also for killing bacteria or viruses. Therefore, MWDT represents a highly promising therapeutic approach for cancer.

MWDT serves as an effective complement to other CRC treatments. Ferroptosis induced by MWDT differs significantly from the cell death caused by traditional therapies (such as chemotherapy, targeted therapy, and immunotherapy). While conventional methods induce regular cell death, particularly apoptosis, therapies that induce apoptosis are prone to developing resistance [3, 39–41]. Mechanistically, ferroptosis is triggered by a lethal accumulation of oxidized phospholipids containing polyunsaturated fatty acids (PUFA), a process distinct from the unique cellular signaling cascades involved in programmed cell death pathways [9]. Therefore, tumor therapies targeting ferroptosis may overcome the resistance observed in traditional therapies [42]. Moreover, compared to chemotherapy and targeted therapy, MWDT reduces damage to normal cells, mitigates side effect risks, and enhances tumor clearance. MWDT does not rely on specific mutations or molecular markers in tumor cells, making it potentially applicable to a broader range of patients, particularly when targeted therapies are ineffective or tumors develop resistance.

Additionally, MWDT-induced ferroptosis in cancer cells alters the components of the tumor microenvironment (TME) and activates or regulates immune responses within the TME [43]. As immunotherapy is gradually becoming an important component of CRC treatment, its efficacy largely depends on the TME. Ferroptotic cancer cells release a variety of immune-stimulating signals [44, 45], enhancing immunogenicity, promoting macrophage polarization to the M1 phenotype, and promoting T cell infiltration and activation within the tumor [13]. Additionally, the primary immunotherapeutic approach in CRC involves regulating immune checkpoint molecules on effector and target cells within the TME [46]. However, this approach often shows poor efficacy in tumors lacking immune cells. MWDT partially overcomes this limitation. Xie et al. recently discovered that in pancreatic ductal adenocarcinoma (PDAC), targeted deletion of PTK2B impairs the differentiation and polarization of monocyte-derived macrophages, remodels the PDAC microenvironment, and enhances the efficacy of anti-PD-1 immunotherapy [47]. Therefore, we hypothesize that the downregulation of PTK2B induced by MWDT may influence CRC immunotherapy by altering the TME. Specifically, MWDT, by modulating the PTK2B/STAT3/GPX4 axis, may promote ferroptosis while activating immune responses, thus offering a potential combination strategy with immunotherapy. Compared to ICB monotherapy, the combination of MWDT may enhance the effectiveness of immunotherapy, especially in tumors where conventional ICB therapy is ineffective.

As a focal adhesion tyrosine kinase, PTK2B is aberrantly expressed in various tumors and is closely associated with tumor initiation and progression. PTK2B promotes tumorigenesis, migration, and invasion by activating several signaling pathways, including Wnt/β-catenin, PI3K/Akt, MAPK/ERK, and TGF-β/EGFR/VEGF, making it a promising target for cancer therapy [48, 49]. Recent studies suggest that PTK2B regulates STING-TBK1 activation by enhancing the polymerization of TBK1 and STING, ensuring an effective innate immune response [50]. Interestingly, we found that overexpression of PTK2B reversed MWDT-induced ferroptosis, highlighting its critical role in regulating this process. This finding underscores the importance of the PTK2B/STAT3/GPX4 pathway in MWDT-induced ferroptosis. Furthermore, the inhibition of PTK2B expression by MWDT further supports the strategy of enhancing ferroptosis in CRC treatment through targeting PTK2B.

There are some limitations to this study. First, while we confirmed the critical role of the PTK2B/STAT3/GPX4 axis in MWDT-induced ferroptosis, we did not explore the specific mechanism by which MWDT reduces PTK2B expression. Future studies need to investigate how MWDT affects PTK2B expression and whether this effect is direct or indirect. Additionally, although we confirmed that MWDT increases intracellular iron levels, the underlying mechanism has yet to be explored. It remains unclear whether MWDT regulates CRC cell iron metabolism by affecting iron uptake, storage, or efflux mechanisms. Future studies should further investigate how MWDT modulates iron metabolism and its role in ferroptosis.

## Conclusions

**Fig. 8:**
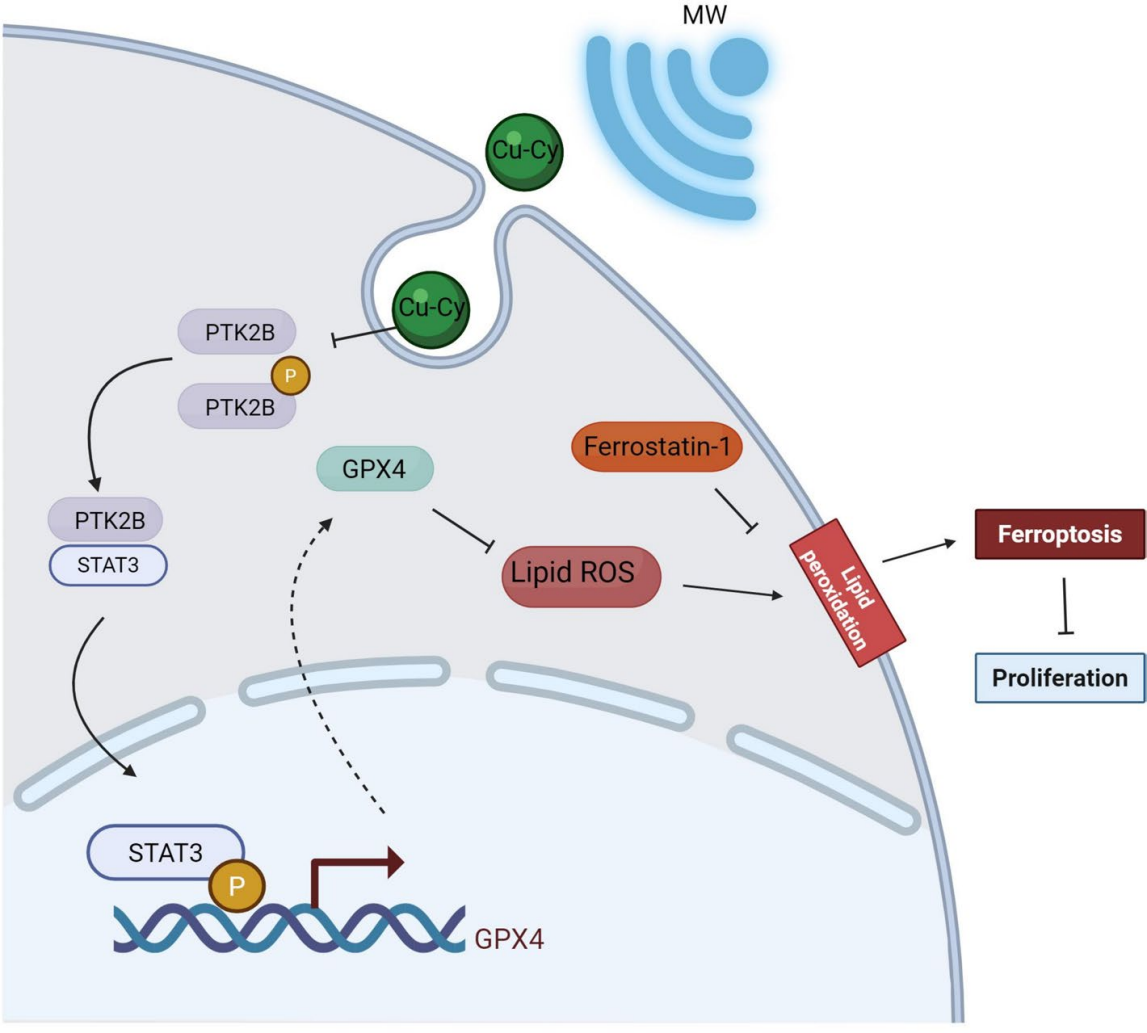
Schematic representation of the MWDT-PTK2B-STAT3-GPX4 axis that synchronically regulates CRC progression. Created with BioRender

In conclusion, our study provides valuable insights into the molecular mechanisms underlying MWDT-induced ferroptosis in CRC cells. By targeting the PTK2B/STAT3/GPX4 axis, MWDT may offer a novel therapeutic approach to overcome chemotherapy resistance and effectively treat CRC (Fig. 8). Future research should explore the in vivo efficacy of MWDT and investigate combination strategies with other ferroptosis inducers to enhance its therapeutic potential. Additionally, understanding the complex molecular interactions within the PTK2B/STAT3/GPX4 pathway is crucial for optimizing ferroptosis-based cancer therapies.

## Materials and methods

### Cell culture and transfection

Human colorectal cancer cell lines HCT15 and HCT116 were obtained from the Type Culture Collection of the Chinese Academy of Sciences (Shanghai, China). Cell identity was verified by short tandem repeat (STR) profiling, and all lines were confirmed mycoplasma-free before use. Cells were maintained in RPMI-1640 medium (Gibco, USA) supplemented with 10% fetal bovine serum (Procell, China) and 1% penicillin–streptomycin in a humidified incubator at 37 °C with 5% CO₂.

To elevate PTK2B expression, an overexpression plasmid (HonorGene, Changsha, China) was introduced using Lipo8000 transfection reagent (Beyotime, China) according to the manufacturer’s protocol. After transfection, overexpression efficiency was assessed by Western blotting.

For stable knockdown, shRNA sequences targeting PTK2B were cloned into the pLKO.1-puro vector. HCT15 and HCT116 cells were infected with lentiviral particles encoding either the PTK2B-shRNA or a non-targeting control (shCon), followed by puromycin selection. Knockdown was confirmed by qRT-PCR and Western blot.

### Cu-Cy-mediated microwave dynamic therapy (MWDT)

Cells were seeded in 6-well plates and allowed to adhere for 24 h (37 °C, 5% CO₂). Culture medium was then replaced with fresh medium with or without 20 μg/mL Cu-Cy, yielding four conditions: control, MW only, Cu-Cy only, and MWDT. After a 24 h incubation with Cu-Cy, the MW and MWDT groups were exposed to microwaves at 2450 MHz, 20 W for 3 min using a radiator probe. Cells were subsequently processed for the indicated assays.

### Lipid peroxidation assay

HCT15 and HCT116 cells (5 × 10^5^cells/well) were plated in 6-well plates and subjected to the designated treatments. After removing the medium and washing with PBS, cells were incubated with C11-BODIPY 581/591 (D3861, Thermo Fisher Scientific, USA) for 30 min in the dark. Following three PBS washes, cells were detached with trypsin, collected, and resuspended in PBS for flow cytometry (NL-3000, Cytek). Data were analyzed with FlowJo v10.6.

### Colony formation assay

Cells were seeded at 1,500 cells/well in 6-well plates. Twenty-four hours later, the indicated treatments were applied, after which the medium was replaced with fresh complete medium. On day 10, colonies were washed with PBS, fixed with 4% paraformaldehyde for 10 min, stained with crystal violet for 10 min, rinsed with PBS, air-dried, and photographed.

### Cell viability assay

For proliferation/viability measurements, 5,000 cells/well were plated in 96-well plates and treated with MWDT ± Ferrostatin-1 (Fer-1, 5 μM) for 24, 48, or 72 h. CCK-8 reagent (10 μL/well) was added and incubated for 2 h, and absorbance at 450 nm was read using a microplate reader (Molecular Devices, Sunnyvale, CA, USA).

### EdU incorporation assay

Cell proliferation was further examined using the BeyoClick™ EdU-488 kit(C0071S, Beyotime, China). Approximately 1 × 10^4^cells were seeded per well in 96-well plates. After attachment, the EdU workflow was carried out strictly following the manufacturer’s instructions, protected from light. Images were captured by a fluorescence microscope.

### Quantitative real-time PCR (qRT-PCR)

Total RNA was isolated using TRIzol(Invitrogen, USA). RNA concentration and purity were determined with a NanoDrop 2000/2000C spectrophotometer. cDNA was synthesized with Takara kit RR0047A, and qPCR was conducted using SYBR® Green Premix Pro Taq HS (AG11701, Accurate Biotechnology, Hunan, China). GAPDH served as the internal control. Relative expression was calculated using the 2⁻ΔΔCt method. All reactions were performed in triplicate.

### Western blotting

Cells were lysed on ice with RIPA buffer (NCM Biotech, China) supplemented with protease inhibitors. Lysates were clarified by centrifugation, proteins were denatured, separated by SDS-PAGE, and transferred to PVDF membranes. After blocking in 5% skim milk in TBST, membranes were incubated overnight at 4 °C with primary antibodies against PTK2B (1:1000, Proteintech), ACSL4 (1:1000, Proteintech), GPX4 (1:1000, Proteintech), and ACTIN (1:1000, Proteintech). After washing, membranes were incubated with HRP-conjugated secondary antibodies (1:5000, 1 h, room temperature), developed with chemiluminescent substrate, and imaged. Band intensities were quantified in Photoshop, and target/ACTIN ratios were used for statistical analysis.

### Malondialdehyde (MDA) measurement

Lipid peroxidation was also evaluated using an MDA assay kit (S0131S, Beyotime). After treatments, cells were lysed and mixed with the kit working solution according to the manual, incubated at 100 °C for 15 min, centrifuged, and 200 μL of supernatant was transferred to a 96-well plate. Absorbance at 532 nm was measured on a microplate reader.

### Immunohistochemistry (IHC)

Paraffin-embedded sections were deparaffinized, rehydrated, and subjected to antigen retrieval. Slides were incubated overnight at 4 °C with primary antibodies against GPX4 (1:200, Proteintech) or PTK2B (1:200, Proteintech). After PBS washes, sections were incubated with appropriate secondary antibodies for 1 h at room temperature. DAB was used for chromogenic detection (approximately 8 min), followed by rinsing with water. Slides were counterstained with hematoxylin(30 s), blued, dehydrated, and mounted with neutral resin. Images were acquired under a light microscope, and staining was quantified as indicated.

### Statistical analysis

Data are presented as mean ± SD (*n* = 3). Statistical analyses were performed in GraphPad Prism 8.0. Two-tailed unpaired t-tests were used for comparisons between two groups; one-way ANOVA was applied for multiple-group comparisons. Differences were considered statistically significant at *P* < 0.05.

## Supplementary Information

Supplementary Material 1.

## Data Availability

The data and materials that support the findings of this study are available from the corresponding author J-C.X. upon reasonable request.
