# Supplementary material for: Microwave dynamic therapy induces ferroptosis in colorectal cancer by targeting PTK2B to regulate STAT3-mediated GPX4 expression
Source: Mol Biomed. 2025 Oct 30;6:91. doi: 10.1186/s43556-025-00322-2 (PMC12575923; doi:10.1186/s43556-025-00322-2)
Supplement: Supplementary file 1 — Supplementary Material 1. [file 43556_2025_322_MOESM1_ESM.docx]

**Supplementary Methods**

**Chromatin Immunoprecipitation (ChIP) Assay**
ChIP assays were performed using the ChIP Assay Kit (Beyotime). First, cells were crosslinked and membrane lysed, followed by nuclease treatment with micrococcal nuclease to fragment the chromatin. The digested and crosslinked chromatin was then incubated with STAT3 antibody (CST). IgG (CST) was used as a positive and negative control, respectively. After thorough washing, DNA was eluted from the magnetic beads and used for qRT-PCR analysis.

**Co-immunoprecipitation (Co-IP) assay**

The CRC cells with 90-100% confluence were seeded in 10 cm plates for endogenous Co-IP, or PTK2B-FLAG plasmid was transfected into HCT116 cells, and Co-IP was performed 48 hours after transfection. The cells were washed three times with ice-cold PBS, scraped off, and collected. The cells were then lysed on ice for 1 hour in modified IP lysis buffer (Beyotime, Shanghai, China) containing protease and phosphatase inhibitors. After centrifugation at 12,000 g for 15 minutes at 4°C to remove cell debris, the supernatant was transferred to a fresh Eppendorf tube. Next, 1-2 µg of antibody (target protein antibody or IgG as a negative control) was added and incubated overnight at 4°C. The following day, 10 µL of protein A/G beads were added, and incubation continued at 4°C with gentle shaking for 2-4 hours. After incubation, the beads were washed three times with modified IP lysis buffer to remove nonspecific bound proteins. The washed beads were resuspended in 2× SDS-PAGE loading buffer and boiled at 100°C for 10 minutes to elute the proteins. Finally, 10 µL of the sample was loaded onto an SDS-PAGE gel, transferred to a membrane, and analyzed by Western blotting to detect the expression of the target protein.

**Immunofluorescence (IF) Staining**

Cells were first fixed with 4% paraformaldehyde at room temperature for 20 minutes. After fixation, cells were washed three times with PBS and incubated with 5% BSA at room temperature for 1 hour to block nonspecific binding. Subsequently, cells were incubated overnight at 4°C with primary antibodies against PTK2B, p-PTK2B, p-STAT3, and STAT3. Detailed information on the antibodies is provided in the supplementary materials. Secondary antibodies conjugated with Alexa Fluor 488 and Alexa Fluor 594 (1:500, Beyotime Biotechnology) were applied to detect the signals. Cells were counterstained with DAPI (Beyotime) for 30 minutes. Finally, fluorescence images were acquired using a Leica SP8 confocal microscope.

**Luciferase Reporter Assay**

The promoter region of GPX4 was first amplified by PCR and cloned into the PGL3-basic vector to construct the reporter gene system. The experiment was performed in triplicates, with 3×10⁴CRC cells seeded in each well of a 24-well plate. After 24 hours of culture, cells were transfected with the PGL3-GPX4/promoter reporter plasmid and Renilla plasmid. After 24 hours, cells were transfected with either STAT3 plasmid or left untreated to assess the effect of STAT3. Finally, according to the manufacturer's instructions, firefly luciferase and Renilla luciferase activities were measured using the Dual-Luciferase Reporter Assay Kit (E1980, Promega, USA) to evaluate GPX4 promoter activity and analyze the regulatory role of STAT3.

### **Protein-Protein Docking Methodology**​

Protein structures of key targets were retrieved from the PDB database (https://www.rcsb.org). Using PyMOL software, the proteins were prepared through dehydration and structural extraction. The processed structures were then subjected to protein-protein docking via GRAMM (https://gramm.compbio.ku.edu/). Resultant complexes were analyzed for chemical bonding interactions using LigPlot+ software, and visualized through structural rendering in PyMOL.

Recombinant Protein Purification from E. coli

The recombinant plasmids pGEX-4T-1-GST-PTK2B-KD (encoding residues 360-690), pGEX-4T-1-GST-PTK2B-KD(K457R), pGEX-4T-1-GST-GFP, and pET28a-His-STAT3 were transformed into Escherichia coli strain BL21(DE3). Recombinant fusion proteins were purified from bacterial lysates using either Ni-Sepharose beads or Glutathione-Sepharose 4B beads according to the manufacturer’s instructions.

In Vitro Kinase Assay

Equal amounts (0.5 μg) of recombinant His-STAT3 protein and GST-tagged PTK2B or its kinase-inactive mutant were mixed in low-salt kinase buffer containing 25 mM Tris-HCl (pH 7.5), 10 mM MgCl₂, and 200 μM ATP. The reaction mixture (30 μL total volume) was incubated at 30 °C for 30 minutes to allow phosphorylation. Reactions were terminated by adding SDS-PAGE loading buffer, followed by heating at 95 °C for 5 minutes prior to SDS-PAGE and subsequent Western blot analysis.

**In vivo xenograft mouse study**

All animal experiments were approved by the Animal Ethics Committee of the Second Xiangya Hospital, Central South University. Five-week-old female BALB/c nude mice were purchased from Hunan Slaike Jingda Experimental Animal Co., LTD. HCT116 cells were resuspended in serum-free medium at a density of 5×10^6^ cells /mL and mixed with the matrix at a 1:1 ratio. A total of 100 μL of cell suspension was injected subcutaneously into 4-week-old female BALB/c nude mice. When the tumor was visible to the naked eye (about 1 week), the mice were divided into 3 groups (5 mice in each group) according to the random table method (No. 1-15) : Control group, MWDT group and MWDT + Fer-1 group. The tumor-bearing mice in MWDT treatment group were intratumorally injected with Cu-Cy solution (1 μg/μL, 30 μL) at 24 h before microwave irradiation. Fer-1 (5 mg/kg) was injected intraperitoneally every 4 days. Tumor size was measured and recorded every 1 day. At the end of the study, mice were euthanized, and tumors were dissected and weighed. Specimens were fixed in neutral formalin.

**Supplementary tables and figures**

**Supplemental Table 1.** The sequences of primers for the qRT-PCR analysis

| **Primers** | **Forward (5’-3’)** | **Reverse (5’-3’)** |
| --- | --- | --- |
| GPX4 | CAGTGAGGCAAGACCGAAGT | GGGGCAGGTCCTTCTCTATC |
| PTK2B | AGATGTGGAAAAGGAGGACG | CTCAGCAGGATGGAGGTGAT |
| GAPDH | GCCAAGGTCATCCATGACAACTTTGG | GCCTGCTTCACCACCTTCTTGATGTC |

**Supplemental Table 2.** The GPX4 primers used in chip assay

| **Primers** | **Forward (5’-3’)** | **Reverse (5’-3’)** |
| --- | --- | --- |
| GPX4 promoter | AATCCAAACCCCTGCCTGTA | CGCGGTATGTGCTCAGAAAA |

**Supplemental Table 3.**

| Antibodies | Product number | Company | County |
| --- | --- | --- | --- |
| β-actin | 66009-1-Ig | Proteintech | China |
| GPX4 | 67763-1-lg | Proteintech | China |
| GPX4 | T56959 | Abmart | China |
| STAT3 | db15725 | Diagbio | China |
| P-STAT3 | [db14015](http://www.diagbio.com/product/PNOdb14015.html) | Diagbio | China |
| PTK2B | sc-393181 | Santa Cruz | American |
| P-PTK2B | sc-293142 | Santa Cruz | American |
| STAT3 | 12640 | Cell Signaling Technology | American |

**Supplementary Figures**


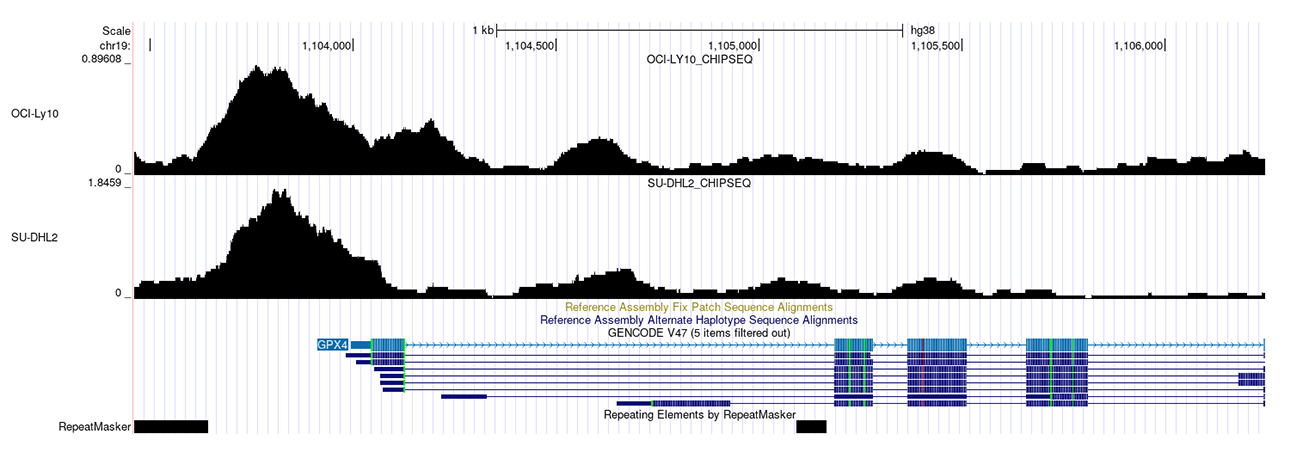


Fig.S1. **STAT3 ChIP-seq signal profile at the GPX4 gene locus by Cistrome Data Browser.**
